# Effects of Mesh Size and Escape Gaps on Discarding in an Australian Giant Mud Crab (*Scylla serrata*) Trap Fishery

**DOI:** 10.1371/journal.pone.0106414

**Published:** 2014-09-02

**Authors:** Matt K. Broadhurst, Paul A. Butcher, Brian R. Cullis

**Affiliations:** 1 NSW Department of Primary Industries, Fisheries Conservation Technology Unit, Coffs Harbour, New South Wales, Australia; 2 National Institute Applied Statistics Research Australia, Faculty of Engineering and Information Sciences, University of Wollongong, Wollongong, New South Wales, Australia; 3 Australia and Computational Informatics, CSIRO, Canberra, Australia; Aristotle University of Thessaloniki, Greece

## Abstract

In response to concerns over excessive discarding from Australian recreational round traps (with four funnel entrances) used to target giant mud crabs, *Scylla serrata*, an experiment was done to assess the independent and cumulative utility of paired, bottom-located horizontal escape gaps (46×120 mm) and increasing mesh size (from 51 to 101 mm). Compared to conventional traps comprising 51-mm mesh throughout, those with the same mesh size and escape gaps caught significantly fewer (by 95%) undersize (<85 mm carapace length – CL) crabs while maintaining legal catches. Traps made from 101-mm mesh (but with the same funnel entrances as conventional designs) and with and without escape gaps similarly retained fewer undersize crabs and also yellowfin bream *Acanthopagrus australis* (the key bycatch species) by up to 94%, but there were concomitant reductions in fishing power for legal sizes of *S. serrata*. Although there were no immediate mortalities among any discarded crabs, there was a greater bias towards wounding among post molts than late inter-molts and less damage to individuals in the 101-mm conventional than 51-mm conventional traps (without escape gaps). The results support retrospectively fitting escape gaps in conventional *S. serrata* traps as a means for reducing discarding, but additional work is required to determine appropriate mesh sizes/configurations that maximize species and size selectivity.

## Introduction

The giant mud crab *Scylla serrata* is among the most popular recreationally targeted crustaceans in northern Australia (from Western Australia to New South Wales–NSW), with an estimated total annual catch exceeding 2.5 million individuals; most (92%) of which are trapped [1], [2]. This total catch is comparatively greater than that of many other local recreational species, however, owing to minimum legal sizes (MLS – interstate variations of ∼130–150 mm carapace width – CW), personal quotas (5–10 per day) and poor trap selectivities, only 32% of all *S. serrata* are harvested [1], [2]. The remaining catches (both adults and juveniles) are discarded, along with largely unknown quantities of incidentally caught teleosts.

Although previous studies have shown that, like other portunids [3], recreationally trapped and discarded *S. serrata* survive with few negative impacts (mostly restricted to limb damage), at least some of the incidentally caught teleosts die, and typically due to within-trap predation [2]. Further, beyond the perception of excessive discarding as being socially unacceptable, there are concerns regarding the so-called ‘ghost fishing’ of poorly selective traps, which if lost can perpetuate unaccounted fishing mortality [4]. Such issues warrant investigating modifications to recreational *S. serrata* traps to improve their species and size selectivity [5], [6].

It is well-established that the selectivity and efficiency of crustacean traps are influenced by various factors, including their: spatial and temporal deployments (relative to biological condition/status of the target species [5], [7]; general design and/or shape [2], [8]; number and/or type of entrances [9], [10]; bait type [11]; presence or absence of escape gaps [5], [6], [12]; and the size [13], [14] and shape of meshes [15]. The importance of some of these variables has been assessed for Australian *S. serrata* traps [2], [5], [6].

For example, in one NSW estuary, Butcher et al. [2] compared hoop nets and round- and rectangular-shaped traps, and like studies on another local portunid (blue swimmer crabs *Portunus pelagicus*) revealed that round traps (∼50-mm stretched mesh opening–SMO) were by far the most efficient design [3], [8]. These traps had four funnel-shaped entrances and caught up to three times as many *S. serrata* and teleosts (mostly yellowfin bream *Acanthopagrus australis*) than the next most efficient trap. However, the 50-mm mesh meant that ∼30% of *S. serrata* were undersized based on NSW legislation (<85 mm carapace length–CL, or ∼130 mm CW).

In an attempt to quantify and improve the selectivity of 50-mm SMO round traps, Rotherham et al. [6] assessed the effects of some of the other factors listed above, including spatial and temporal (diel) deployments, two or four entrances, and the presence or absence of four sizes of paired (located at opposite sides of the traps), horizontal rectangular escape gaps (55×85, 55×95, 45×85 and 45×95 mm). Notwithstanding some spatial and temporal inconsistencies in performances, the traps with four entrances caught fewer *A. australis* and legal-sized *S. serrata* than those with two entrances, while those fitted with escape gaps (irrespective of size) similarly caught fewer yellowfin bream and undersized *S. serrata* than conventional designs [6]. The authors recommended ongoing work with more suitable escape-gap sizes within the tested range (e.g. 55×90 mm). However, in another study on the smaller cogeneric orange mud crab *Scylla olivacea*, Jirapunpipat et al. [12] observed that for a constant vertical opening, there was a positive correlation between the horizontal length of gaps and the escape of undersize crabs and non-target species. Intuitively, because the passage of crabs through escape gaps should be dictated by their maximum carapace depth [16] the length of the opening would seem less important, and perhaps could be maximized.

A third study in the Northern Territory also supports the concept of closely aligning the height of escape gaps to the carapace depth of *S. serrata* [5]. For 50-mm SMO round traps with two entrances, the authors showed that two sizes of paired escape gaps (42×120 mm and 50×120 mm) were effective in reducing catches of *S. serrata* ≥130 mm CW (∼85 mm CL) and ≥150 mm CW (∼100 mm CL) by up to 20 and 40%, respectively, although there were spatial performance differences attributed to size distributions and inter-specific antagonistic encounters (e.g. small crabs were more likely to escape from traps in the presence of larger individuals). Compared to controls, traps with both sizes of escape gaps caught up to four times fewer fish [5].

While the above studies show that retrospectively fitting escape gaps (irrespective of their dimensions) to *S. serrata* traps is a simple, potentially cost-effective strategy for improving size and species selectivity, perhaps a first coherent step in such a process for any net-based fishing gear is to determine the most appropriate mesh size for the targeted species [17]. Doing so ensures complete coverage of ‘escape’ openings across the entire gear, and not just at one or two locations (like escape gaps). However, despite the regulation of a minimum legal mesh size of 50 mm in NSW (clearly much smaller than the MLS of 85 mm CL or ∼130 mm CW), no formal studies have been done to investigate the effects of mesh size on the selectivity or efficiency for *S. serrata*, or key bycatches.

One potential issue associated with increasing mesh size throughout the entire trap is a confounding effect on entrances; a factor known to affect efficiency [9], [10], [14]. Such impacts might be circumvented by maintaining the same entrance (including mesh size), and restricting mesh changes to the trap frame (sides, top and bottom) only. In terms of selecting an appropriate mesh size, as a starting point, this should be dictated by the morphology (maximum carapace height, width and depth) of the smallest legal-sized *S. serrata*, which corresponds to a SMO of ∼100 mm [2]. This mesh size has a perimeter matching the maximum girth of a 250-mm TL *A. australis* [18], which co-incidentally is also their MLS, and a size larger than that of most individuals typically entering portunid traps [2], [3], [6].

Given the above, the main aim in this study was to compare the effects on size and species selectivity and trap efficiency associated with increasing mesh size and/or using wider than previously assessed escape gaps (46×120 mm) in NSW. A secondary aim was to test the hypothesis that none of these technical changes (or their combination) negatively affected the injury and/or immediate mortality of discards.

## Materials and Methods

### Ethics statement

The experiment was done in the Corindi River system (29°59′S 153°13′E) and within the Solitary Islands Marine Park; approval for which was granted by the NSW Marine Parks Authority (permit number 2012/003) and under the Department of Primary Industries scientific collection permit (No. P01/0059(A)-2.0). Animal ethics approval was issued by the NSW DPI Animal Care and Ethics Committee (Ref. 03/12). None of the work involved endangered or protected species.

### Study location

**Figure 1:**
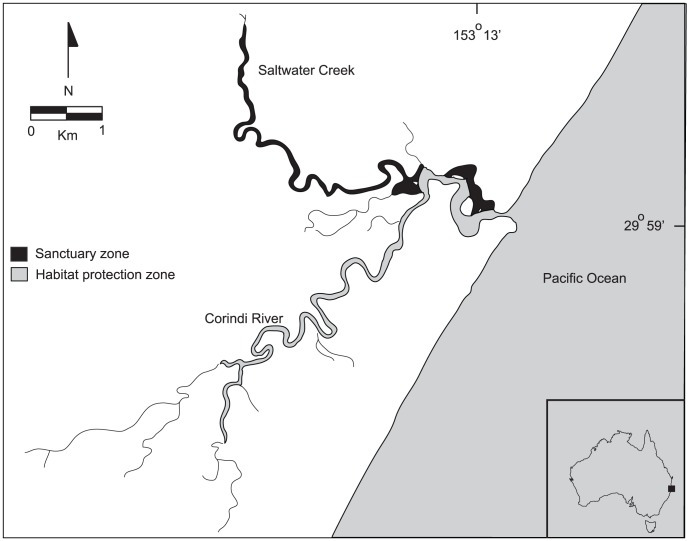
Map of the area sampled.

Two areas within the Corindi River system were selected for the research (Figure 1). The first area (termed ‘sanctuary zone’) had been closed to all fishing effort for ten years and was chosen to ensure sufficient numbers of adult *S. serrata* (Figure 1). The second area (‘habitat protection zone’) was open to recreational fishing and, therefore, comprised proportionally more juveniles/subadults as a consequence of regular discarding (Figure 1).

### Traps

**Figure 2:**
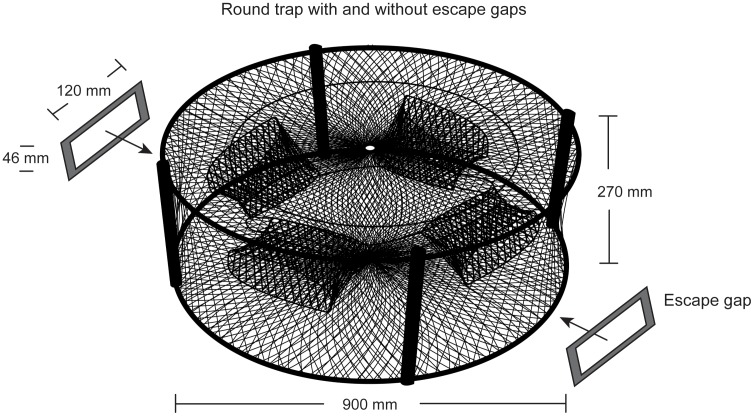
Generic round trap with and without escape gaps.

Seven replicates of four treatment traps were constructed. All traps comprised orange knotted polyethylene (PE) mesh stretched across two 0.90 m diameter–Ø galvanized steel rings (9-mm Ø rod) separated by four plastic vertical stanchions (270 mm) and had four 300×200 mm semi-closed funnel entrances made from nominal 51-mm SMO PE mesh [2] (Figure 2). The four treatments only differed in their frame mesh size (nominal 51 or 101 mm) and the presence or absence of two escape gaps, and were termed the: (1) ‘51-mm conventional’; (2) ‘51-mm escape-gap’; (3) ‘101-mm conventional’; and (4) ‘101-mm escape-gap’ traps. The escape gaps were rectangular polyvinyl chloride frames (46×120 mm) located immediately perpendicular to the base of each trap and opposite each other, and matched the maximum carapace depth of legal-sized *S. serrata* (Figure 2). Fifteen replicate meshes were measured (SMO to the nearest 1 mm) in each trap using a local, purpose-built gauge.

On each fishing day, the 28 traps (seven replicates of each treatment) were baited with ∼0.8 kg of chopped sea mullet *Mugil cephalus* in a 250-×200-mm wire-mesh bait bag (10-×10-mm mesh) and attached to a rope and 100-mm Ø buoy. The replicate traps were randomly deployed in the morning (typically between 07:30 and 11:30) across mangrove-lined mud and sand channels (5–50 m wide and 0.6–4.5 m deep), and 50 m apart to ensure independence [2] and left undisturbed for 24 h. During retrieval, replicate water temperature (°C) and salinity (PSU) were recorded (using an Horiba U10 water meter) at approximately every fifth trap. Over seven weeks, a total of 20 daily deployments were done (between Monday and Friday), with 8 and 12 in the habitat protection and sanctuary zones, respectively.

### Relative trap efficiencies and immediate impacts to all discards

After trap retrieval, all catches were immediately counted by species and assessed for mortality. All teleosts were measured for total length (TL to the nearest 0.1 cm) before being assessed for fin damage (present or absent) and discarded. *S. serrata* were emptied into the boat, sexed, measured with vernier calipers (to the nearest 0.1 cm) for CL, CW and carapace depth (CD), and their molt stage determined [19]: (1) post-molt — clean and highly flexible shell, no wear on chelae; (2) early inter-molt – moderately flexible shell and some wear on chelae; or (3) late inter-molt – little or no flex in shell, and/or large, significant wear on chelae.

The locations and numbers of missing and/or damaged limbs (cheliped, pereopod or swimmeret) and/or any carapace damage and when this occurred (i.e. during the deployment, removal from traps or measuring) were quantified and each wound was categorized as ‘sealed’ or ‘unsealed’ following Ulhmann et al. [20]. All *S. serrata* were t-bar tagged [2] and released at their capture site before the trap was rebaited and set at a different, randomly located site.

### Data analyses

The hypothesis of no differences in mesh sizes between the various traps was tested using a linear model (LM). Linear regressions of CL against CW and CD were fitted separately for male and female *S. serrata* and then compared using analyses of co-variance (ANCOVA). The remaining data were analyzed in generalized linear mixed models (GLMM) to test the null hypothesis of no differences among treatment traps in (1) catches of *S. serrata* and (2) the key teleost bycatch, and (3) new damage to discarded *S. serrata* and (4) when it occurred (i.e. during trap deployment, removal or measuring).

Because all *S. serrata* were tagged and released, with many subsequently recaptured on multiple occasions (see Results), instead of using individuals as the experiment unit describing catches (and therefore defaulting to trap used each day as the observational unit), we considered the individual crab per trap per day as the experimental and observational unit and the response variable given by the binary outcome of caught or not caught. This approach implicitly exploits multiple recaptures and therefore allows for a direct ‘preference’ analysis of ‘trap type’ for *S. serrata*. We use the term ‘catchability’ here, and in subsequent modelling we allowed for multiple captures by including a so-called random ‘frailty effect’ for each *S. serrata*. Such an approach assumes the conditional independence of catchability given the set of fixed and random linear predictors. Specifically, we fitted a GLMM with a binary distribution and logit link. The GLMM included trap type and crab-specific covariates of ‘sex’, ‘molt stage’ (post and early and late inter-molts) and CL as fixed effects. Environmental fixed effects included the water ‘depth’, ‘temperature’ and ‘salinity’, while ‘days’, ‘locations’, replicate ‘traps’ and the interaction between traps and days were fitted as random terms in the GLMM.

The interactions between sex, molt stage and CL, and trap type were also explored through inclusion as fixed effects, but discarded from the final model unless significant (*p*<0.05). To examine the catchability response profiles for each trap type, we followed an approach motivated by Holst and Revill [21], but instead of using polynomial approximations, cubic smoothing splines (CSS) were applied following Verbyla et al. [22]; who illustrated how CSS may be fitted within the framework of LMMs or GLMMs.

The remaining GLMMs assessing the numbers of key teleost bycatch (*A. australis*–which were not tagged; see Results) and new damage to discarded *S. serrata* and when it occurred were analyzed considering the trap used each day as the experimental unit. For *A. australis* catches, in addition to trap type, the fixed effects considered included depth, temperature and salinity, while the random terms were traps, days and locations. For the GLMM assessing damage to discarded *S. serrata*, we included the same fixed and random effects (and interactions) as for crab catchability above, but with an additional random term of tag number. The GLMM for when damage occurred comprised the same random and fixed effects, but no interactions for the latter, owing to the sparse data.

All models were fitted in ASReml-R [23] using either Poisson (wounding among *S. serrata* and yellowfin bream catches using a log link) or binomial (when wounding to *S. serrata* occurred using a logit link) distributions. The statistical significance of fixed terms was determined using approximate Wald *F*-tests. Significant differences among categorical factors (such as trap type and molt stage) were explored using the Benjamini–Hochberg–Yekutieli procedure to control the false discovery rate (FDR) [24].

## Results

The 101-mm traps had a significantly larger mesh than the 51-mm designs (LM and FDR, *p<*0.01) but there were no significant differences between traps with the same nominal mesh sizes, with means ±SE of 50.92±0.07 and 101.34±0.07 mm, respectively (LM and FDR, *p*>0.05). All traps were deployed across comparable mean ±SD water depths (1.87±0.71 and 1.49±0.48 m) and temperatures (17.15±1.01 and 17.77±1.58°C, respectively at each location) although salinities were somewhat divergent (22.97±9.25 and 32.96±6.75).

### Relative efficiencies

Catches comprised 178 teleosts and 552 individual *S. serrata* (46–121 mm CL); of which 241 crabs were re-caught (some up to seven times) for a total catch of 863 (including 157 undersize) (Table 1). Most crabs were early inter- (48% of the total) followed by late inter-molts (37%) and post molts (15%) (Table 1).

**Table 1:** Summary of catches and their treatment from the four types of traps (n = 7) deployed across 20 days in two areas of the Corindi River.

			Traps			
Variable			51-con	51-escape	101-con	101-escape
*Scylla serrata*	Total no. caught		376	223	147	117
	No. of legal size		266	218	114	104
	Proportion of total no. undersized		0.29	0.02	0.22	0.11
	No. of recaptured individuals		92	77	41	31
	No. of recaptures		128	102	47	33
	Mean CL (SD) of total caught		14.30 (2.19)	15.37 (1.41)	14.62 (1.86)	14.89 (1.75)
	Sex ratio (M∶F) of total caught		2.23∶1	2.98∶1	1.92∶1	2.16∶1
	Molt stages of total caught	Post-molt	62	23	17	31
		Early inter-molt	176	73	61	104
		Late inter-molt	138	50	39	88
	Removal method	Emptied	336	204	142	115
		Pulled	40	19	4	2
	Removal time (SD)		4.88 (8.79)	4.01 (6.78)	2.78 (4.58)	2.38 (2.74)
	Damage (limbs)	Yes	44	14	5	5
		If yes, mean (SD) number of limbs	1.25 (0.61)	1.21 (0.58)	1.00 (0.00)	1.00 (0.00)
Bycatch	*Acanthopagrus australis*	No.	115	34	12	6
		Mean total length in mm (SD)	17.25 (3.57)	17.13 (3.97)	18.11 (3.35)	24.35 (1.30)
	No. of *Platycephalus fuscus*		0	0	1	0
	No. of *Girella tricuspidata*		1	0	1	0
	No. of *Anguilla australis australis*		5	2	0	0
	No. of *Epinephelus coioides*		0	0	0	1
	No. of *Monodactylus argenteus*		0	1	0	0

51-con, 51-mm conventional trap; 51-escape, 51-mm escape-gap trap; 101-con, 101-mm conventional trap; 101-escape, 101-mm escape-gap trap.

ANCOVA failed to detect significant differences in coefficients between regressions of CL and either CW or CD (*p*>0.05), but elevations were significantly divergent, with males slightly higher and wider than females for any given CL (*p*<0.01). Separate regressions were calculated as CD = 0.5529CL−0.3403 (*r^2^* = 0.98) and CW = 1.4683CL+3.6171 (*r^2^* = 0.98) for females and CD = 0.5513CL+0.5774 (*r^2^* = 0.97) and CW = 1.4370CL+7.7331 (*r^2^* = 0.98) for males, which, at the MLS of 85 mm, meant that giant mud crab had carapaces that were 46.65 and 47.44 mm high and 128.42 and 129.88 mm wide, respectively.

**Figure 3:**
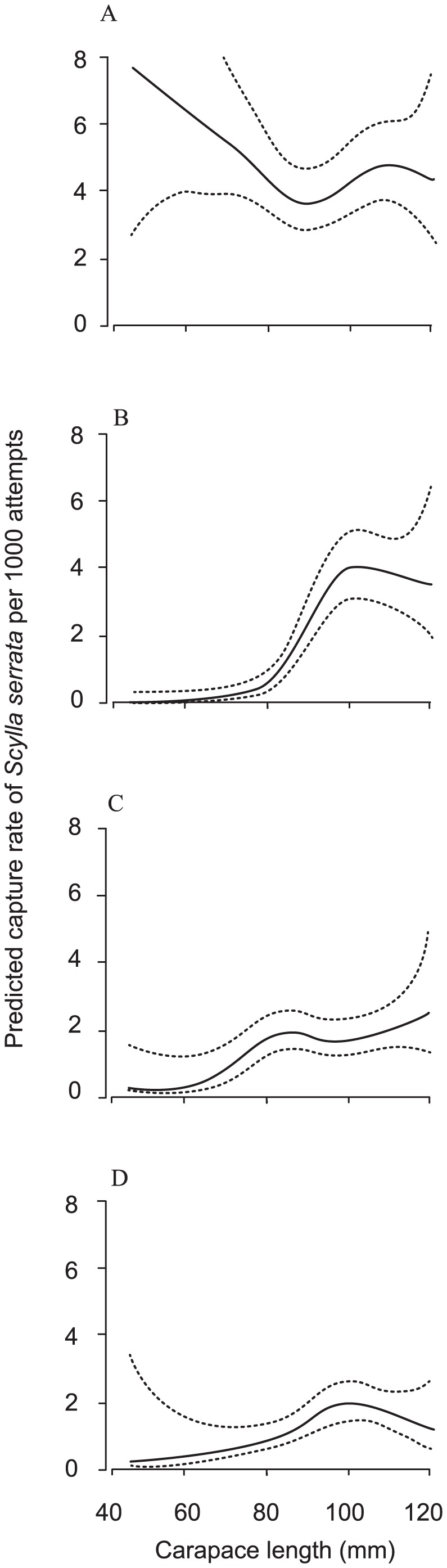
Fitted response profiles (solid lines) and associated approximate 95% coverage intervals of predicted capture rates (per 1000 attempts) against carapace length (mm) for the (A) 51-mm conventional, (B) 51-mm escape-gap, (C) 101-mm conventional and (D) 101-mm escape gap traps.

The catchability of *S. serrata* was negatively correlated to salinity, biased towards males and varied among the four trap designs according to their CL (Figure 3, Table 2, GLMM, *p*<0.01). For the CL and trap type interaction, the response curves revealed that compared to all other treatments, the 51-mm conventional trap retained proportionally more smaller and fewer larger *S. serrata*, while the 51-mm escape-gap showed the opposite, attaining a catch rate of about 4 crabs per 1000 attempts (Figure 3a, b). The 101-mm conventional and 101-mm escape-gap traps also caught few smaller crabs, but only increasing to approximately 2 individuals per 1000 for those >∼80 and 90 cm CL, respectively (Figure 3c, d).

**Table 2:** Summary of fixed factors and Wald *F*-values in parsimonious generalised linear mixed models explaining variability in the catchability of *Scylla serrata* and numbers of *Acanthopagrus australis* among four treatment traps (51-mm conventional, 51-mm escape-gap, 101-mm conventional and101-mm escape-gap traps), and subsequent damage to *S. serrata* and when this occurred (during trap removal or measuring).

Fixed factor	df	Trap catchability for *S. serrata*	No. of *Acanthopagrus australis*	Wounding among *S. serrata*	When wounding occurred
Depth	1	2.36	2.50	5.64*	0.03
Temperature	1	0.05	1.33	0.77	0.26
Salinity	1	8.70**	0.20	0.04	0.03
Sex	1	10.14**	Na	2.18	1.39
CL	1	Na	Na	0.22	0.06
Molt stage	2	0.84	Na	6.93*	0.54
Trap type (T)	3	Na	23.47***	14.64**	1.04
CL × T	3	22.65***	Na	Na	Na

Depth, temperature and salinity describe the water at the location of the traps. Na, not available for testing or not appropriate for presentation. df, degrees of freedom. *p<0.05, **p<0.01, ***p<0.001.

**Figure 4:**
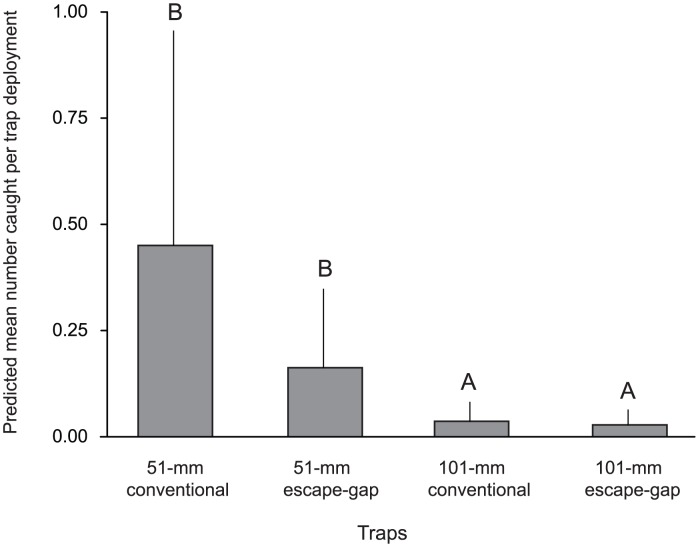
The predicted mean ±SE numbers of *Acanthopagrus australis* in each of the four treatment traps per deployment. Dissimilar letters indicate significant differences detected in false-discovery-rate pairwise comparisons (*p*<0.05).

The only teleost caught in sufficient quantities for analyses was *A. australis* (80–350 mm TL), with a maximum of 11 individuals in any one trap. Catches were significantly affected by trap type (GLMM, *p*<0.05; Figure 4, Table 2) with FDRs showing no significant differences within traps of the same mesh size, either with or without escape gaps (*p*<0.05), but significantly fewer fish in the 101- than the 51-mm traps (*p*>0.05; Figure 4). There were too few data to analyze the differences in sizes, although like for *S. serrata*, proportionally larger *A. australis* were caught in the 101-mm than the 51-mm designs (Table 1).

### Impacts to discards

No *S. serrata* were dead during discarding, but 68 individuals had new damage, which was typically restricted to one missing limb and mostly either a swimmeret or cheliped (Table 1). Wounding was significantly affected by water depth (positively), molt stage and trap type (GLMM, *p*<0.05; Table 2). False-discovery-rate pairwise comparisons revealed that across all four trap designs, post molts were significantly more damaged (predicted mean of 0.091±0.027 individuals per trap deployment) than late inter-molts (0.042±0.012 individuals per trap deployment; *p*<0.05). However, there was no significant difference in damage rates between early inter-molts (0.043±0.011 individuals per trap deployment) and *S. serrata* in the other two molt-stages (FDR, *p*>0.05).

Irrespective of molt stage, crabs incurred significantly less wounding in the 101-mm conventional (predicted mean ±SE of 0.03±0.01 individuals per trap deployment) than the 51-mm conventional traps (0.13±0.02 individuals per trap deployment; FDR, *p*<0.05); perhaps because they were more quickly and more frequently emptied rather than pulled (Table 1). However there were no other significant pairwise differences between the conventional and the 51-mm escape-gap (0.06±0.02 per trap deployment) and 101-mm escape-gap traps (0.04±0.02 per trap deployment) (FDRs, *p*>0.05). All wounds were sealed and only one individual was damaged during the actual trap deployments (which was subsequently removed from analyses), with the rest wounded either during trap removal (56 individuals) or measuring (12); but with no significant bias towards any of the measured fixed effects (GLMM, *p*>0.05; Table 2).

Three *A. australis* were dead during discarding: one from the 51-mm escape-gap trap and two from the 51-mm conventional trap. All three individuals had tissue loss, suggesting predation. The remaining discarded teleosts all swam vigorously away; with their physical damage limited to slightly frayed caudal fins.

## Discussion

The study reaffirms the utility of simple, rectangular escape gaps for improving the size and species selectivity of traps targeting *Scylla* [5], [12] and crustaceans in general [25], [26]. It is also clear that while increasing mesh size was comparably effective at improving selectivity for both *S. serrata* and *A. australis*, there was a concomitant reduction in the catchability of the assessed traps for the former. These observations can be discussed by considering the morphology and possible behavior of *S. serrata*, and to a lesser extent, *A. australis*, during trap ingress and escape.

The size of the escape gap was chosen to allow undersized *S. serrata* (85 mm CL in NSW) to pass through. Further, based on the morphology of *A. australis* [18] the escape gaps were also sufficient to allow a <130-mm TL individual to swim out in a vertical orientation, and a 350-mm TL individual (the largest size caught–in the 51-mm conventional trap) to squeeze through horizontally; a behavior previously observed for several species, including sparids [27]. Depending on the location in the trap, the 101-mm mesh also should have allowed some small *S. serrata* and up to a 250-mm TL *A. australis* to escape.

While both modifications appear to have independently and cumulatively facilitated the escape of undersize crabs and some smaller *A. australis*, the increase in mesh size had a broader impact that affected fishing power. More specifically, either more *S. serrata* escaped across a greater range of sizes, or, more likely, fewer individuals had a preference for entering either of the 101-mm mesh traps.

Some previous studies have demonstrated a negative relationship between mesh size and the fishing power of both crustacean [13] and fish traps [28], but especially the latter [25]. This correlation has been attributed to either (1) malleability in morphology, effectively allowing some organisms to penetrate smaller than expected mesh perimeters; or (2) changes in visual [28], [29], and possibly tactile stimuli that reduce ingress. Both hypotheses could account for the relatively lower catches of *A. australis* in the 101-mm traps however, despite *S. serrata* comprising different molt stages none were soft enough to facilitate mesh ingress or escape larger than their measured morphology. It is more likely that fewer crabs entered the 101-mm mesh traps through the funnels.

No work has been done to assess the behavioral responses of *S. serrata* to baited traps, although studies have been done with other portunids [9], [10], [30]. For example, Asian paddle crabs *Chaybdis japonica* were observed to typically approach baited traps from downstream and, after contacting the meshes, pushed their dactyls through in repeated attempts at gaining access to the food [9], [10]. Failing to penetrate, they either gave up or moved laterally around the trap perimeter until encountering the funnel entrances. Comparable results have been observed for other crustaceans (including portunids), with individuals often moving away after repeated failure at trap entry [30], [31]. If *S. serrata* responded similarly here, then they would have been able to penetrate (their chelipeds) more easily and further between the 101-mm meshes, and may have spent relatively more time attempting to gain access, instead of searching for a funnel entrance. The extent of such behavior is likely to be species- and/or gear-specific, since at least some other studies have failed to detect similar reductions in fishing power associated with increasing mesh for other crustaceans, including portunids [7], [32].

Although not as efficient, the 101-mm conventional trap caused relatively less damage to *S. serrata* than the 51-mm conventional design. An earlier study showed that most damage to trapped *S. serrata* occurred when swimmerets were impinged in meshes [2]. Possibly, larger and fewer meshes meant that crabs could more easily fall out; evidenced by significantly faster release from the 101-mm traps. However, all wounds were sealed and so notwithstanding some potential impacts on defenses and prey acquisition (e.g. associated with lost chelipeds) any protracted mortality was probably minimal [20]. This hypothesis is supported by the quantity and frequency (up to seven times) of recaptured crabs in traps; many within consecutive days of release.

While three *A. australis* died (presumably due to predation) in the 51-mm mesh traps, there were no fatalities in the larger-mesh designs. It is likely that any threatened small fish could escape simply by passing through the larger meshes; and probably did so frequently given the significant reductions in catches by these traps. By contrast, *A. australis* in a 51-mm escape-gap trap would need to swim towards the bottom (where crabs were probably orientated) and risk confrontation while attempting to escape through the openings. Such impacts would have been further exacerbated by the relatively greater abundances of *S. serrata* in the smaller-meshed traps.

Assuming that the reduction in fishing power for *S. serrata* in the 101-mm traps was caused by the tactile and/or visual stimuli discussed above, it might be advantageous to assess an intermediary mesh size. As part of this work, video footage would be beneficial. Alternatively, composite-mesh traps [27] could warrant consideration, with small mesh around the sides but larger mesh on the base and top, so that fish can swim upwards or downwards during fishing and retrieval, respectively. One consideration is using the same traps to target the smaller-sized *P. pelagicus* at different locations and times. The 46-mm high escape gaps assessed here would allow many legal *P. pelagicus* (≥60 mm CL) to escape. Rather, a vertical opening of 31–34-mm probably would be more appropriate. It might be feasible to have variable escape-gap inserts and some compromise on mesh size. For example, ∼75-mm mesh might still retain legal-sized blue swimmer crabs [7], while allowing some teleosts to escape.

Because trap usage is likely to increase in response to the relatively lower associated unaccounted fishing mortality and also fuel intensity than many other gears, detailed studies describing their performances and mechanisms to maximize species and size selectivity are warranted. While the current gears have few impacts, it is apparent that simple changes like inexpensive, retrospectively fitted escape gaps could considerably improve selectivity. Mandating the use of such modifications seems a justifiable precautionary approach.
